# Supplementary material for: Development of a computer-aided design software for the quantitative evaluation of aesthetic damage
Source: PLoS One. 2019 Dec 18;14(12):e0226322. doi: 10.1371/journal.pone.0226322 (PMC6919621; doi:10.1371/journal.pone.0226322)
Supplement: S1 Code site — 1. (ZIP) [file pone.0226322.s001.zip › [EESC_jr][Projeto]Relatorio_07_21/reports/templates_zip/medialoot-lumino-admin-bootstrap-template.zip!/Lumino Admin Bootstrap Template/lumino/index.html]

Lumino - Dashboard


Toggle navigation

LuminoAdmin

- User 
  - Profile
  - Settings
  - Logout

- Dashboard
- Widgets
- Charts
- Tables
- Forms
- Alerts & Panels
- Icons
- Dropdown
  - Sub Item 1
  - Sub Item 2
  - Sub Item 3
- Login Page

2. Icons

# Dashboard

120

New Orders

52

Comments

24

New Users

25.2k

Page Views

Site Traffic Overview

#### New Orders

92%

#### Comments

65%

#### New Users

56%

#### Visitors

27%

Chat

- **John Doe** 32 mins ago

  Lorem ipsum dolor sit amet, consectetur adipiscing elit. Nulla ante turpis, rutrum ut ullamcorper sed, dapibus ac nunc. Vivamus luctus convallis mauris, eu gravida tortor aliquam ultricies.
- **Jane Doe** 6 mins ago

  Mauris dignissim porta enim, sed commodo sem blandit non. Ut scelerisque sapien eu mauris faucibus ultrices. Nulla ac odio nisl. Proin est metus, interdum scelerisque quam eu, eleifend pretium nunc. Suspendisse finibus auctor lectus, eu interdum sapien.
- **John Doe** 32 mins ago

  Lorem ipsum dolor sit amet, consectetur adipiscing elit. Nulla ante turpis, rutrum ut ullamcorper sed, dapibus ac nunc. Vivamus luctus convallis mauris, eu gravida tortor aliquam ultricies.

Send

To-do List

- Make a plan for today
- Update Basecamp
- Send email to Jane
- Drink coffee
- Do some work
- Tidy up workspace

Add
